# Biomarkers in Lysosomal Storage Diseases

**DOI:** 10.3390/diseases4040040

**Published:** 2016-12-17

**Authors:** Joaquin Bobillo Lobato, Maria Jiménez Hidalgo, Luis M. Jiménez Jiménez

**Affiliations:** 1 Servicio de Bioquímica Clínica, Unidad de Gestión Clínica de Laboratorios, Hospital Universitario Nuestra Señora de Valme, 41014-Sevilla, Spain; 2 Servicio de Fisiopatología Celular y Bioenergética, Servicios Centrales de Investigación, Universidad Pablo de Olavide, 41013-Sevilla, Spain; mariajihid@hotmail.com (M.J.H.); lmjimenezj@telefonica.net (L.M.J.J.)

**Keywords:** biomarkers, lysosomal storage disorders (LSDs), enzyme replacement therapy (ERT)

## Abstract

A biomarker is generally an analyte that indicates the presence and/or extent of a biological process, which is in itself usually directly linked to the clinical manifestations and outcome of a particular disease. The biomarkers in the field of lysosomal storage diseases (LSDs) have particular relevance where spectacular therapeutic initiatives have been achieved, most notably with the introduction of enzyme replacement therapy (ERT). There are two main types of biomarkers. The first group is comprised of those molecules whose accumulation is directly enhanced as a result of defective lysosomal function. These molecules represent the storage of the principal macro-molecular substrate(s) of a specific enzyme or protein, whose function is deficient in the given disease. In the second group of biomarkers, the relationship between the lysosomal defect and the biomarker is indirect. In this group, the biomarker reflects the effects of the primary lysosomal defect on cell, tissue, or organ functions. There is no “gold standard” among biomarkers used to diagnosis and/or monitor LSDs, but there are a number that exist that can be used to reasonably assess and monitor the state of certain organs or functions. A number of biomarkers have been proposed for the analysis of the most important LSDs. In this review, we will summarize the most promising biomarkers in major LSDs and discuss why these are the most promising candidates for screening systems.

## 1. Introduction

The continuous recycling of macromolecular components is essential for the functional integrity of cells. This macromolecular rejuvenation depends not only on permanent biosynthesis, but also on the degradation of these macromolecules [1] which takes place through the hydrolytic process in specific subcellular compartments—the lysosomes—discovered by Christian de Duve [2]. Henry Hers [3] first identified the specific lysosomal enzyme deficiency (acid-α-glucosidase) responsible for glycogen storage disease type 2, also called Pompe disease [1]. This discovery allowed for the development of the concept of lysosomal storage disorders (LSDs), of which more than 70 have now been described.

These disorders are the result of deficient enzymatic function, which may be due to alterations in lysosomal hydrolases, transporter proteins, membrane proteins, or activator proteins—all of which are related to lysosomes. The loss of any of these functions leads to a progressive accumulation of one or more metabolic precursors within lysosomes [4], causing irreversible cell dysfunction and damage to multiple organs [5].

The incidence of individual LSDs varies between 1 in 20,000–100,000 live births. The overall incidence of all LSDs is at least 1 in 5000–10,000 live births [1]. Further, a newborn screening study for Fabry disease alone revealed an unexpectedly high incidence of 1 case in approximately 3100 newborns [6].

Characteristically, the phenotype of LSDs is present as a continuous spectrum from the most serious childhood forms to the milder adult forms. This diversity in the manifestation of the same disease is due to the different degrees of deficiency of the enzyme involved, and therefore the speed at which the substrates are accumulated. Although most LSDs lack effective treatments (even in cases of timely detection), in some cases this diagnosis offers the opportunity to establish early therapeutic measures in order to intervene before irreversible damage occurs in the affected organs. The refinement of enzyme replacement therapy (ERT), the emergence of small molecule therapies, and the promising developments of gene therapy have resulted in a leading role for the diagnostic process and its relevant biomarkers.

## 2. Biomarkers

In 2001, the Study Group of National Institutes of Health (NIH) defined a biomarker as a characteristic which can be measured and evaluated objectively as an indicator of a biological or pathological process or a pharmacological response to a treatment. In our case, this “characteristic” is a biochemical compound [7].

An ideal biomarker must have certain specific characteristics:

(a) The biomarker should be easy to quantify in an accessible clinical substance (serum, plasma, urine, etc.) which reduces the need for invasive procedures (cerebrospinal fluid, tissue biopsy, etc.). It should be possible to quantify the concentration or activity level of the biomarker reliably, quickly, cheaply, and reproducibly.
(b) Concentrations or activity of the biomarker must not be subject to wide variations in the general population.
(c) The biomarker should ideally reflect the impact of the disease at all sites where it is manifest, not only at a select few sites.
(d) To facilitate diagnosis, the biomarker should be specifically altered (elevated or reduced) in the relevant disease and unaffected by unrelated conditions. There should be a distinction in the prevalence of the biomarker in untreated patients and control subjects, and the prevalence should vary in response to treatment.

These characteristics are summarized in Table 1.

## 3. Role of Biomarkers in the Study of LSDs

The diagnosis of LSDs usually begins by directly measuring the enzyme activity responsible for the development of each disease. Simultaneously, the determination of other more-or-less specific biomarkers in different biological samples (blood, urine, cerebrospinal fluid) can be carried out, contributing to diagnosis. Other options used are histochemical and/or ultrastructural studies in different samples obtained from organs or tissues (bone marrow, liver, muscle, skin, etc.), providing evidence for the presence of characteristic cells or the accumulation of specific non-metabolized products, which may even affect their structures and/or functions.

The increase in therapeutic options available for LSDs has radically transformed their prognosis and patient quality of life. Enzyme replacement therapy (ERT)—consisting of the intravenous (i.v.) administration of the recombinant functional enzyme—has been applied to many LSDs, such as Pompe disease, type I Gaucher disease, Fabry disease, and Mucopolysaccharidoses (MPS) types I and II. Even though ERT is one of the most successful treatments available for LSD patients, it is not without limitations [8]. Substrate reduction therapy (SRT) acts by restricting the biosynthesis of metabolites upstream of the malfunctioning catabolic pathway, partially inhibiting the synthesis of the defective hydrolase substrate in order to reduce substrate influx into the catabolically compromised lysosome, thus limiting its storage. Alternatively, enzyme enhancement therapy (EET, also known as pharmacological chaperone therapy) can enhance the residual activity of the defective lysosomal enzyme due to its stabilization. Both of these strategies hold great potential, since they should result in a better bio-distribution than the recombinant enzyme. Further, they employ low-molecular-weight molecules that can be taken orally and cross the blood-brain barrier [8]. Hematopoietic cell transplantation (HCT) from healthy compatible donors has been widely used to treat many diseases, including LSDs. Its “little sister”—gene therapy—involves manipulation of the genetic information of the diseased cells to correct a genetic defect or to give cells a new feature that allows them to overcome the pathological alteration.

To assess the efficiency of these therapies, it is necessary to use biomarkers that allow us to analyze (with scientific rigor) the evolution of the disease over time, determining how the deposits of accumulated products are diminishing. In addition, biomarkers are useful for adding evidence to the study of specific enzyme activity.

Biomarkers are a key component of not only the diagnosis of LSDs, but also for monitoring patients and choosing the best therapeutic option in each case. The European Medicines Agency (EMEA), in collaboration with the European Federation of Pharmaceutical Industries and Associations (EFPIA), organized a joint workshop on Biomarkers on 15 December 2006, in which it stated that biomarkers are the key in the shift away from the “one fits all” to the “right drug at the right dose in the right patient group” approach. Hence, biomarkers play an important role for scientists and industry in drug development and for regulators in the approval process [9]. Thus, biomarkers play a critical role in current developments towards novel therapeutic approaches and in pharmacogenetics.

Another advantage of the use of biomarkers over techniques for the quantification of enzyme activity is the existence of pseudo-deficiency. For example, it is common in both Pompe and MPS I for a change in the genetic sequence to occur that results in decreased enzymatic activity, but not enough to cause the disease. Patients with only one pseudo-deficiency allele do not tend to develop the disease, nor do those with either a pseudo-deficiency allele and a mutated allele. This phenomenon greatly jeopardizes confidence in a diagnosis that relies on the enzymatic activity that the individual presents. It is in these cases where biomarkers play a major role (if the gene sequencing is not available).

Among the biomarkers used for diagnosis and/or monitoring of LSDs, there is none that can be described as a “gold standard”. They may be clinical or biological, and may be useful for a majority of LSDs or may be specific to a select few [10].

One of the ways in which biomarkers for LSDs are classified stems from the origin of that biomarker. Some are substrates that accumulate in cells as a consequence of the pathological enzyme defect, while others are the (sometimes atypical) “products” of cytopathological processes (Table 2).

For these reasons, recent study of biomarkers has generated considerable interest. In this review, we provide a summary of the most promising biomarkers for several LSDs, chosen for their prevalence and therapeutic potential following evaluation in pilot studies, and those already included in national and international newborn screening programs [11].

### 3.1. Fabry Disease

The enzyme deficiency of α-galactosidase A (GLA; EC 3.2.1.22) results in Fabry disease (FD; OMIM 301500), a disorder with a prevalence at birth of 0.22/100,000 [12], with sex-linked recessive inheritance and caused by mutations in the *GLA* located on chromosome X (Xq22.1). The enzyme deficiency causes the lysosomal storage of globotriaosylceramide (Gb3), among other glycosphingolipids.

The accumulation in the tissues of non-degraded substrates—mainly at the smooth muscle cells and endothelial cells level—ultimately results in the symptoms of the disease: angiokeratomas, intolerance to heat, hypo/anhydrosis, micro-albuminuria, neuropathic pain (acroparesthesias), and corneal opacity (cornea verticillata), amongst others. The progression of the disease eventually leads to progressive renal impairment, heart diseases, or cerebrovascular disease (stroke) [13,14].

Surprisingly, many female heterozygotes also exhibit symptoms of FD due to the Lyon effect of random X-inactivation [15].

Diagnosis of these patients may also be less than straightforward. The GLA enzyme activity assays enable the identification of affected males relatively easily, but in the case of heterozygous females, an overlap occurs that makes the clear classification of these patients difficult. This limitation, coupled with the difficult goal of implementing ERT in FD [16,17], has led to the search for early diagnostic biomarkers that have been incorporated into the habitual study of the disease.

As stated previously, the substrate of the deficient enzyme Gb3 has been the traditional biomarker since early studies showed that its concentration is high in Fabry patients [18]. This accumulated globoside is excreted by cells, and can be quantified in body fluids as plasma and urine [11]. However, several studies have shown that there is not a clear correlation between the Gb3 levels and the clinical manifestation or severity of the disease [19,20].

Using experience in the diagnosis of other glycosphingolipidoses, researchers deduced (before it could be detected and quantified) the existence of a water-soluble deacylated metabolite of Gb3—the globotriaosylsphingosine (lysoGb3) in FD [13,21]. Later, it was discovered that lysoGb3 could be quantified and studied in these patients following the development of suitable techniques of liquid chromatography and tandem-mass spectrometry (LC-MS/MS) [22].

LysoGb3 is not only a better marker to show a higher elevation of concentrations than Gb3, but a better correlation was also achieved with the classical clinical manifestations of disease [23]. In addition, the overlap effect observed for FD females disappears, although the total concentration is lower than in FD males.

Despite the great progress that these findings have led to, the biomarker is not without limitations. Researchers have been unable to detect significant elevations in plasma lysoGb3 in samples from males with atypical mutations of the disease [13]. The clearest example is the p.N215S mutation, which produces a cardiac form of Fabry disease. New advances in metabolomics have overcome these limitations with the recent discovery of Lyso-Gb3 analogues [24] and methylated/non-methylated Gb3 isoforms [25] in plasma and urine (the non-methylated isoform increased in patients with the p.N215S mutation).

The recently-developed technology of mass tandem spectrometry and new detection protocols that are beginning to emerge in the wake of the discovery of these new biomarkers [26] seems to predict an encouraging future for knowledge about the disease’s progression, genotype/phenotype relationship, and best treatment models.

### 3.2. Gaucher Disease

Gaucher disease (GD) is a relatively common glucosphingolipidoses caused by deficient activity of the lysosomal enzyme glucocerebrosidase (GBA; EC 3.2.1.45). This causes a lysosomal accumulation of its substrate, glucosylceramide. GD pathogenesis can be also explained by defects in enzyme conformation, endoplasmic reticulum stress, defects in calcium homeostasis (one of the mechanisms responsible for the neuropathology observed in patients with acute neuropathic GD), increased sensitivity to oxidative stress, and changes in autophagy mechanisms [27].

Gaucher disease has been divided into three clinical types based on its primary neurological involvement and disease progression:

GD type 1 or its non-neuropathic form (OMIM 230800) has a prevalence of 1/100,000 [12], and is characterized by haematological, visceral, and bone involvement, as well as an absence of primary brain involvement. The early onset of symptoms (in the first or second decade of life) can predict the progression rate and severity of the disease [28,29].

In the type 2 (or acute neuropathic GD) form (OMIM 230900), there is neurological involvement from birth and rapid brain deterioration. The median age of death is nine months [28,30]. Its prevalence is very low (0.01/100,000 in Europe) [12].

GD type 3 has a prevalence of 0.05/100,000 [12], and is the chronic neuropathic form (OMIM 231000). Its clinical manifestations begin in childhood and adolescence. The central nervous system (CNS) may be affected, but usually there is no effect at the bulbar level. It is the most complex form of the disease because symptoms and signs vary in intensity, which determine various subgroups of the disease.

Glucosylceramide accumulation is especially significant in macrophages. This phenomenon is believed to be due to the degradation of exogenous lipids derived from the renewal of blood cells. These glucosylceramide-laden macrophages are the typical Gaucher cells which can be seen in bone marrow aspiration with a May–Grünwald–Giemsa or Hematoxylin–Eosin staining.

Although both the presence of Gaucher cells and the concentration of glucosylceramide may seem to be the most logical biomarkers, they have important limitations—Gaucher cells cannot be used to quantify involvement or disease progression clearly, and there may be an overlap in glucosylceramide concentration in affected and unaffected patients [31].

Chitotriosidase (ChT) has long been considered the classic biomarker for GD. This enzyme has been shown to correlate with various clinical parameters, and its concentration is used to monitor and adjust the treatment of patients. It is specifically expressed by human phagocytes, including macrophages and neutrophils, and indirectly reflects the total body burden of Gaucher cells. Different tests use the chitinase activity of the enzyme to activate chromogenic or fluorogenic products and quantify their activity [32].

In the general population, there are a significant number of individuals (1:20) with poor chitotriosidase activity due to a polymorphism consisting of 24 bp duplication of the *CHIT1* gene. Individuals with G102S substitution do not react to the substrate standard tests, methylumbelliferyl-4-chitotrioside. Although there are different solutions to these problems [31], this difficulty prompted researchers to search for alternative biomarkers.

The pulmonary and activation-regulated chemokine (CCL18/PARC) is also secreted by Gaucher cells, and its concentration is 20 to 50 times greater in affected patients [33]. This is the chosen marker when there are polymorphisms affecting chitotriosidase levels.

Currently, the biomarker glucosylsphingosine proposed by Rolfs et al. [34] has emerged as a more effective alternative to chitotriosidase and CCL18. Their results show that the concentration of glucosylsphingosine in Gaucher samples was about 100 times higher than in healthy patients and in patients with other LSDs. By using LC-MS/MS, the study achieved 100% specificity in identifying Gaucher patients.

Recently incorporated plasma biomarkers for GD are macrophage inflammatory protein 1-alpha and 1-beta (MIP-1α and MIP-1β) [35]. Together with cathepsin K [36], these biomarkers are specifically used for the study of bone disease in these patients—a major complication with severe consequences in terms of morbidity.

In addition, metabolic-enzymatic alteration in these individuals causes (1) increased anabolism of glucosylceramide ganglioside in the patient’s cells, leading to increased ganglioside GM3 (monosialodihexosylganglioside) concentrations; and (2) activation of an alternative metabolic pathway that produces glucosylsphingosine—a characteristic by-product [13] quantifiable by LC-MS/MS—a metabolite that stands out as a very promising biomarker.

A final and promising biomarker for GD is osteopontin, a sialoprotein of the extracellular matrix involved in cell adhesion processes and bone metabolism, with levels increased in various pathologic processes. Quantified using Luminex xMAP^®^ technology, osteopontin provides an alternative to chitotriosidase, and seems to overcome its limitations. Its concentration is increased in untreated adult GD type 1 patients, and rapidly falls after receiving ERT. It appears to be more sensitive than chitotriosidase, varying more rapidly and more drastically (82% versus 52% reduction) [27]. However, current studies have been limited in scope, and further investigation is warranted.

The higher prevalence of GD as compared to other LSDs has led to a greater number of well-designed studies for this condition, and consequently, a greater number of findings on biomarkers of clinical relevance.

### 3.3. Krabbe Disease

Krabbe disease (globoid cell leukodystrophy) (KD; OMIM 245200) is a devastating autosomal recessive neurometabolic condition caused by lysosomal galactocerebrosidase deficiency (GALC; EC 3.2.1.46), responsible for cleavage of galactosyl moieties from its four substrates: galactosylceramide, lactosylceramide, lactosylsphingosine, and psychosine [37]. It is a rare (1/100,000 prevalence) [12] and demyelinating pathology where non-degraded substrates accumulate in the central and peripheral nervous systems with severe psychiatric and neuromotor consequences. The disease manifests itself in different clinical forms, depending on the patient’s age at onset; the infantile form is the most serious and common.

The main tests used for diagnosis are neuroimaging, neurophysiological studies, cytochemistry, cerebrospinal fluid (CSF), and histological analysis of skin and peripheral nerves.

Recently, psychosine (PSY, galactosylsphingosine) has been suggested to possibly serve as an indicator of the presence and/or progression of Krabbe disease [38]. It can be quantified in blood, dried blood spot (DBS), and cerebrospinal fluid. Its relevance is enhanced by the fact that it is a direct toxic agent that can kill many cell types, including oligodendrocytes and Schwann cells. Thus, a close—although not yet clarified—relationship exists between the concentrations of these cells and the development of pathophysiological damage of disease.

Psychosine concentrations from diagnosed infantile patients are at least four times higher than asymptomatic new-born children, and nearly an order of magnitude greater than healthy new-born children [39].

Several methods have been described to measure PSY, albeit most with low efficiency. The system designed by Orsini and colleagues [39] is noteworthy for quantifying PSY in DBS by liquid chromatography tandem mass spectrometry (LC-MS/MS). The main problem with the method—already solved with the use of ultra-performance liquid chromatography tandem mass spectrometry (UPLC-MS/MS)—was that it was not possible to separate and identify psychosine from glucosylsphingosine independently.

Separating psychosine from glucosylsphingosine is not currently believed to be absolutely necessary, as psychosine is supposed to increase in Krabbe patients from birth, but glucosylsphingosine increases in Gaucher patients later [31,39].

An algorithm can be developed, since a high concentration of psychosine plus glucosylsphingosine combined with low GALC activity would suggest Krabbe disease, whereas the same result with a low acid β-d-glucosidase (GBA) activity would suggest Gaucher disease. Further study of the use of psychosine as a biomarker of Krabbe disease is necessary [40].

The most optimistic data suggest that this method could at times replace neuroradiology or neurophysiological studies in some medical centers used in the current follow up protocol of many hospitals.

### 3.4. Mucopolysaccharidoses

Mucopolysaccharidoses (MPS) are caused by deficiency of specific enzymes that catalyze the step-wise degradation of glycosaminoglycans (GAGs; mucopolysaccharides), with a total incidence of approximately 1 in 25,000 live births.

There are 11 known enzyme deficiencies involved in the catabolism of chondroitin sulfate (CS), dermatan sulfate (DS), heparan sulfate (HS), and keratan sulfate (KS). Any enzyme deficiency will block GAG degradation and result in the accumulation of GAG macromolecules with a specific carbohydrate or sulfated carbohydrate residue in multiple tissues, resulting in severe dysfunction [41]. The most important data for mucopolysaccharidoses are summarized in Table 3.

Most clinical signs and symptoms for MPS patients do not appear immediately after birth, but instead progress with age. Although the symptoms and severity of MPS vary between individuals and between MPS subtypes, the average life span in most patients is one to two decades if left untreated [41].

Diagnosis of MPS is achieved by three sequential steps. First, GAG detection in urine (where they are readily excreted due to their high polarity) is carried out via a spectrophotometric method with dimethylmethylene blue (DMB), which determines total GAG relative to creatinine in the patient and compares it with an age-matched reference range. If the result is positive, it is followed by a chromatographic separation of the intact GAG (usually by gel electrophoresis), and then by band staining to identify the specific GAG that appears to be elevated in concentration [42]. The most likely subtype of MPS exhibited in the patient can be identified in this way. Finally, demonstration of the specific enzyme defect in DBS, leukocytes, or cultured cells is performed.

There are different techniques used to measure GAGs [43], but tandem mass spectrometric methods seem to be the most promising. However, the direct quantification of GAGs by this process is currently quite an involved process, meaning a major drawback. This limits its utility for the screening of large numbers of samples in screening systems [44]. It is also an expensive technology, and therefore not accessible to most laboratories. It is for this reason that the DMB technique for GAGs determination continues to have its role and advantages in patient screening processes, since it is a simple technique with a short analysis time (no more than 30 min) that is very economical.

Relatively large studies have reported the analysis of glycosaminoglycan fragments by MS/MS as a potential biomarker for mucopolysaccharidoses [45,46].

Recently, a new quantitative UPLC–MS/MS method for HS, DS, and CS has taken advantage of equipment that is available at some clinical laboratories with basic triple quadrupole MS/MS systems to quantify acylcarnitines, amino acids, and other valuable disease biomarkers [42]. The method was able to detect and quantify CS, DS, and HS in the urine and cerebrospinal fluid (CSF) of unaffected individuals and establish an age-specific reference interval of GAG in urine. Thus, the method allows us to determine the urinary levels of these biomarkers and facilitate diagnosis for patients with MPS I, II, III, IVA, and VI, as well as in other lysosomal storage disorders.

In an even more current development, Langereis et al. adapted previous protocols of GAGs quantification by adding the identifying KS [47] so that it not only provides a multiplex assay for the diagnosis of MPS, but also for Mucolipidoses II and III.

As for other conditions, proteomic studies have allowed for the discovery of further potential biomarkers of interest. Heywood et al. [48] obtained favorable results in their studies of β-galactosidase, Collagen type Iα, fatty-acid-binding-protein 5, nidogen-1, cartilage oligomeric matrix protein, insulin-like growth factor binding protein 7, and protein Heg1 in urine. These markers are associated with impaired extracellular matrix, endothelial function, and lysosomal hypertrophy, which are key pathological features of MPS. They demonstrate a relationship between marker concentrations and disease severity, such that the usefulness of these markers lies in their ability to determine the severity of the disease at initial diagnosis—a tremendously important feature. It will be necessary to follow up on these new discoveries.

### 3.5. Niemann–Pick Disease

The term “Niemann–Pick disease” (NP) includes a heterogeneous group of lysosomal lipid accumulation diseases with clinical, biochemical, and molecular features.

In 1961, Crocker classified them into four subgroups (types A–D) [49]. In types A and B (OMIM 257200, 607616; prevalence of 0.25 and 0.4/100,000 [12] respectively), the accumulation of sphingomyelin in different organs and tissues is due to a deficit of acid sphingomyelinase enzyme (ASM; EC 3.1.4.12) caused by mutations in the sphingomyelin phosphodiesterase-1 gene. In types C and D (OMIM 257220, 1/100,000 prevalence) [12] however, a defect in cellular lipid transport can result in several pathological forms of the disorder [50].

We now know that NP-C disease is clinically, biochemically, and genetically distinct from types A and B. The NP-D disease is an allelic variant of type C found only in patients in Nova Scotia (Canada).

As in most lysosomal storage disorders, the clinical spectrum of Niemann–Pick disease A/B is increasingly regarded as a continuum from the most severe to relatively mild presentation [51].

Acid sphingomyelinase plays a major role in sphingolipid metabolism, because it catalyzes the hydrolysis of sphingomyelin to ceramide and phosphorylcholine. Ceramide and related products (such as sphingosine-1-phosphate) are involved in a variety of molecular and cellular processes, and play a central role in a growing number of human diseases [52]. The ASM deficiency in Niemann–Pick disease types A and B causes the accumulation of sphingomyelin and other lipids in various tissues, mainly in the monocyte–macrophage system.

Until recently, sphingomyelin was the only biomarker available for the disease. Although its levels have been shown to be elevated more than 10-fold in the livers and spleens of NP-B patients, levels in plasma have been found to overlap those of normal controls [53].

More promisingly, we determined that lysosphingomyelin (lyso-SPM) levels in DBS from NP-B patients were substantially elevated when compared to normal controls, with no overlap in values [40,53]. The mean lyso-SPM concentration in affected patients was approximately five times higher than in non-affected patients. However, lyso-SPM concentration did not correlate with the amount of residual ASM activity in DBS or with patient age, indicating a need for further study of biomarkers of NPD types A and B.

Niemann–Pick type C, on the other hand, is a neurodegenerative lipid storage disorder caused by dysfunction in one of the lysosomal proteins: NPC1 or NPC2. These proteins are responsible for displacing free cholesterol from the lysosome to other cellular compartments. Dysfunction resulting from mutations in either *NPC1* or the *NPC2* leads to cholesterol accumulation in the lysosome, thereby damaging several organs and is characterized by progressive neurological deterioration [54].

The current diagnostic method for NPD-C is a set of consecutive tests, including chitotriosidase assay and filipin staining of free cholesterol in fibroblasts. However, these methodologies lack sensitivity and specificity [55,56].

Overcoming the limitations of these biomarkers, it was discovered that higher levels of cholestane-3β,5α,6β-triol (C-triol) and 7-ketocholesterol (7-KC) are present in the plasma of NP-C patients when compared to plasma from patients with other lysosomal storage disorders or control subjects [57]. Additionally, these authors showed that plasma concentrations of C-triol and 7-KC are directly correlated with the disease state, and are specific biomarkers for NP-C [58].

Promising results have also been obtained by the study of 24(*S*)-hydroxycholesterol (24(S)-HC), the major polar metabolite by which the brain removes cholesterol. The concentration of this metabolite is decreased in the plasma and CSF of NP-C patients as a reflection of inefficient cholesterol homeostasis in the CNS [59]. Mechanisms that allow for the reduction in lysosomal cholesterol storage also result in an increase of 24-(*S*)-HC levels in plasma and CSF. Its quantification by LC-MS/MS methods has been validated [60], and monitoring it in the clinical trials of new drugs (e.g., in the study of 2-hydroxypropyl-beta-cyclodextrin) [61] has made it possible to assess the efficacy of the results obtained.

Recently, Mazzacuva et al. described a rapid method to quantify novel bile acids in biological fluids (plasma, DBS, and urine) and evaluated their diagnostic potential in NP-C. Two new compounds—NPCBA1 (3β-hydroxy,7β-*N*-acetylglucosaminyl-5-cholenoic acid) and NPCBA2 (probably 3β,5α,6β-trihydroxycholanoyl-glycine) accumulated to a greater degree in NP-C patients. Median plasma concentrations of NPCBA1 and NPCBA2 were 40 and 10 times higher in affected patients than in controls, respectively [62].

The use of animal models for different phases of clinical trials has also proven successful in identifying other relevant biomarkers. One of these is Calbindin D, a compound found in cerebrospinal fluid that has been validated as a biomarker to track disease progression and serve as a supporting outcome measure of therapeutic efficacy. In the feline NPC1 model, high levels of calbindin D were observed to decrease during administration of a treatment. In patients with NPC1, CSF calbindin concentration was significantly higher than in CSF collected from unaffected patients. Additionally, after treatment with SRT, calbindin levels significantly decreased in CSF compared with pre-treatment concentrations [63].

Finally, one study claimed to have found a novel highly sensitive and specific biomarker in plasma for NPC1—lyso-sphingomyelin-509 [64]. Its structure is likely very similar to lyso-SPM, but so far the exact structure has not been elucidated. The authors quantified the biomarker using HPLC-MS/MS, and reported a sensitivity of 100.0% and specificity of 91.0%, with a cut-off of 1.4 ng/mL. Although these results are extraordinary, we consider it appropriate to be cautious and wait for further studies to corroborate its true effectiveness.

### 3.6. Pompe Disease

Pompe disease (PD; OMIM 232300, with a prevalence at birth of 0.81/100,000 [12])—also known as glycogen storage disease type II, or acid maltase deficiency—is a lysosomal storage disorder in which mutations in the *GAA* cause deficiency of acid α-glucosidase enzyme (GAA; EC 3.2.1.20). This leads to the accumulation of glycogen in lysosomes of several tissues and cell types, particularly cardiac, skeletal, and smooth muscle cells [65]. Although glycogen breakdown also takes place outside the lysosome, interruption to lysosomal level affects several key processes, among which is autophagy, a critical survival mechanism in conditions of nutrient deprivation and cellular protein turnover [66].

Cardiac pathology is a characteristic symptom of classic infantile Pompe disease—the most severe form. Usually, it presents in the first few months of life, and infants normally die within the first year. Late-onset forms of Pompe disease involve a wide age range (children, youth, and adults). This latter form initially presents hypotonia, muscle weakness, motor delay, and subsequent respiratory insufficiency [67,68]. The highly variable clinical presentation of the disease with respect to age of onset, disease severity, organ involvement, and clinical course is attributed to its allelic heterogeneity. In addition, it has been shown that there are polymorphisms which act as genotype modulators of PD that might determine the severity of final phenotype [69].

Diagnosis of Pompe disease presents significant difficulties due to the rarity of the condition and the nonspecific nature of its phenotypic characteristics. Presentation in its infantile form is easily recognizable by its characteristic hypotonia, generalized weakness, and cardiomegaly. Patients with the adult form presentation develop less easily-identifiable symptoms that may be difficult to fit into the context of the disease.

Because it is a disease of accumulation in which the pathophysiological damage is progressive, a delay in diagnosis entails organic deterioration that may become increasingly severe and even irreversible [70]. It is therefore important to establish a correct diagnosis as soon as possible and start treatment immediately, especially given that it is one of the diseases for which ERT has proven highly effective. Unfortunately, early identification can be difficult to attain, and up to 20% of patients experience a diagnostic delay of one to five years [70].

GAA hydrolyzes α,1–4 and α,1–6 links of glucose polymers, and has an optimum activity at a pH between 3.7 and 4.5, depending on the substrate concentration. In a typical assay, it is necessary to measure various fractions of GAA activity to establish a specific diagnosis.

Although enzyme diagnosis has a firm scientific basis, this approach is not useful for monitoring disease progression once ERT is implemented. In this case, it is more efficient to use a biomarker resulting from glycogen digestion and other ramified glucose polymers, the tetra-saccharide glucose (Glc4) [71].

Although Glc4 is not specific to PD and its use for diagnostic purposes may be limited in certain aspects, the predominant hypothesis at present considers it to be the product of intravascular degradation of glycogen release from affected muscle tissue in different pathological processes [72], converting it into a tracking biomarker of undeniable utility [73].

Quantification of the compound is carried out in urine samples using a variety of different methodologies: immunoassays, chromatography affinity with specific antibodies, high-performance liquid chromatography with ultraviolet (HPLC/UV) or electrochemical (HPLC/ED) detection, ultra-performance liquid chromatography with mass spectrometry detection (UPLC-MS/MS), etc. Of these, HPLC/UV detection [72,73] is the most widely used technique, due to its efficiency and availability in laboratories.

The correlation between urinary excretion of Glc4 and the response of target tissues to therapy has been previously demonstrated [73]. Thus, patients with the best clinical response to treatment maintain the lowest levels of Glc4. The use of this biomarker is especially relevant in the context of a disease where there is considerable variation in the clinical response to treatment of patients [74].

Other markers currently being studied for their ability to monitor response to treatment of these patients include myostatin and insulin-like growth factor I (IGF-I) [75]. These are serum markers involved in muscular metabolism in growth and regeneration functions. It appears that PD patients have decreased levels of these markers, but are significantly elevated after ERT (129% for IGF-I and 74% for myostatin), reaching levels equivalent to those of control subjects.

## 4. Chronology

Because of the great clinical heterogeneity of these diseases, it is impossible to establish a universal timetable for their clinical and biological control. However, some general recommendations can be applied in various therapeutic circumstances to streamline the monitoring of patients with respect to their individual clinical characteristics and circumstances. Of course, these are recommendations that should be adapted to each specific situation.

When biomarkers are used only to assess the natural history of the disease, they should be analyzed at the time of diagnosis, every three or six months during childhood, and every six months after puberty. It is always necessary to consider the characteristics of the biomarker. For example, the monitoring of hematocrit or platelets in Gaucher disease is not the same as the glycosaminoglycans in urine for mucopolysaccharidoses.

When biomarkers are used to assess response to treatment, they should be assessed prior to the initial administration and whenever there is a change in treatment or the patient’s clinical condition.

When treatment and progression of the disease are stabilized, ongoing assessments can be performed with a frequency ranging from six months during childhood to one year in adults.

Different guidelines have been published that serve as an educational resource for confirmatory testing and subsequent clinical management of pre-symptomatic individuals suspected of having a lysosomal storage disorder. They also help to define a research agenda for longitudinal studies such as the American College of Medical Genetics/National Institutes of Health Newborn Screening Translational Research Network [76].

## Figures and Tables

**Table 1:** Characteristics of the ideal biomarker.

■ Easy to quantify in accessible clinical material ■ Measurements are reliable, quick, reproducible, and cheap ■ Abundance not subject to wide variation in the general population ■ Reflects total burden of disease at all sites ■ Expression specifically altered (elevated or reduced) in the relevant disease ■ Unaffected by unrelated conditions and co-morbid factor ■ Expression increased in the established disease without overlap between untreated patients and healthy subjects ■ Variation of their concentration or activity in response to treatment which is closely correlated with established the clinic-pathological parameters of the disease

**Table 2:** Classification of biomarkers according to the nature of origin.

LSD	Biomarkers	
	Accumulated Substrate	Indirect Products
Fabry disease	Globotriaosylceramide (Gb3)	- Globotriaosylsphingosine (LysoGb3) - LysoGb3 analogs - Methylated/non-methylated Gb3 isoforms
Gaucher disease	Glucosylceramide	- Chitotriosidase (ChT) - Pulmonary and activation-regulated chemokine (CCL18/PARC) - Macrophage inflammatory protein 1-alpha and 1-beta (MIP-1α and MIP-1β) - Cathepsin K - Ganglioside GM3/monosialodihexosylganglioside - Glucosylsphingosine - Osteopontin
Krabbe disease	Galactosylceramide	- Galactosylsphingosine/psychosine
Mucopolysaccharidoses	Dermatan sulfate Heparan sulfate Keratan sulfate Chondroitin-6-sulfate Chondroitin-4,6-sulfate Hyaluronic acid	- Glycosaminoglycan fragments - Others: β-galactosidase, Collagen Iα, fatty-acid-binding-protein 5, nidogen-1, cartilage oligomeric matrix protein, and insulin-like growth factor binding protein 7, Protein HEG1
Niemann–Pick disease	Sphingomyelin Free cholesterol (in fibroblasts)	- Lysosphingomyelin (Lyso-SPM) - Cholestane-3β,5α,6β-triol (C-triol) and 7-ketocholesterol (7-KC) - 24(*S*)-hydroxycholesterol - NPCBA1 (3β-hydroxy,7β-*N*-acetylglucosaminyl-5-cholenoic acid) and NPCBA2 (probably 3β,5α,6β-trihydroxycholanoyl-glycine) - Calbindin D - Lyso-sphingomyelin-509
Pompe disease	Glycogen	- Tetrasaccharide glucose (Glc4) - Myostatin - Insulin-like growth factor-I (IGF-I)

**Table 3:** The most important data in mucopolysacharidoses.

Type	Title	OMIM	Prevalence (/100,000) [12]	Enzyme Deficiency		GAGs Accumulated
MPS I-H	Hurler syndrome	607014	8 P 0.82 BP	Alpha-l-iduronidase	IDUA	DS, HS
MPS I-S	Scheie syndrome	607016		Alpha-l-iduronidase	IDUA	DS, HS
MPS I-HS	Hurler–Scheie syndrome	607015		Alpha-l-iduronidase	IDUA	DS, HS
MPS II	Hunter syndrome	309900	6.7 P 0.68 BP	Iduronate-2-sulfatase	IDS	DS, HS
MPS III-A	Sanfilippo syndrome A	252900	0.5 P 1.4 BP	*N*-sulfoglucosamine sulfohydrolase	SGSH	HS
MPS III-B	Sanfilippo syndrome B	252920	0.09 P	*N*-alpha-acetylglucosaminidase	NAGLU	HS
MPS III-C	Sanfilippo syndrome C	252930	-	Heparan acetyl-CoA: alpha-glucosaminide *N*-acetyltransferase	HGSNAT	HS
MPS III-D	Sanfilippo syndrome D	252940	-	*N*-acetylglucosamine-6-sulfatase	GNS	HS
MPS IV-A	Morquio syndrome A	253000	-	Galactosamine-6-sulfate sulfatase	GALNS	KS, C6S
MPS IV-B	Morquio syndrome B	253010	-	Beta-galactosidase	GLB1	KS
MPS VI	Maroteaux–Lamy Syndrome	253200	0.16 BP	*N*-acetylgalactosamine-4-sulfatase or Arylsulfatase B	ARSB	DS
MPS VII	Sly syndrome	253220	0.01 P	Beta-glucuronidase	GUSB	DS, HS, C4,6S
MPS IX	Hyaluronidase deficiency	601492	-	Hyaluronidase	HYAL	HA

Abbreviations: P: Prevalence; BP: Prevalence at birth; DS: Dermatan sulfate; GAG: glycosaminoglycan; HS: Heparan sulfate; KS: Keratan sulfate; C6S: Chondroitin-6-sulfate; C4,6S: Chondroitin-4,6-sulfate; HA: Hyaluronic acid.
